# Effect of Tai Chi versus aerobic exercise on blood pressure in prehypertension patients (TCOBPP): a study protocol for a 12-month single-blind randomized controlled trial

**DOI:** 10.1186/s13063-022-06840-6

**Published:** 2022-12-12

**Authors:** Xinye Li, Yonghong Gao, Min Wu, Dawei Wei, Xingjiang Xiong, Yan Yang, Yuchen Jiang, Xiandu Pan, Ran Zhao, Fan Yang, Jiahao Sun, Shengjie Yang, Li Tian, Linqiang Ban, Xingye Li, Peifen Chang, Yanwei Xing

**Affiliations:** 1 Beijing University of Chinese Medicine, Beijing, 100029 China; 2 Guang’anmen Hospital, China Academy of Chinese Medical Sciences, Beijing, 100053 China; 3 Key Laboratory of Chinese Internal Medicine of the Ministry of Education, Dongzhimen Hospital Affiliated to Beijing University of Chinese Medicine, Beijing, 100029 China; 4 Dongzhimen Hospital Affiliated to Beijing University of Chinese Medicine, Beijing, 100029 China

**Keywords:** Tai Chi, Aerobic exercise, Prehypertension, Blood pressure

## Abstract

**Background:**

Compared with optimal blood pressure (BP), the prehypertension increases the risk of incident hypertension, cardiovascular (CV) events, and death. Moderate intensity of regular physical activity can reduce BP. However, aerobic exercise has some limitations. As a safe, low-impact, enjoyable, and inexpensive form of exercise that requires minimal equipment and space, Tai Chi is expected as a viable alternative to aerobic exercise. The study aimed to assess the effect of Tai Chi intervention program, compared with aerobic exercise, on the BP in prehypertension patients.

**Methods:**

This study is a 12-month, two-center, single-blind, parallel, randomized controlled trial. Three hundred forty-two patients with prehypertension [with a systolic blood pressure (SBP) in the range of 120 mmHg to 139 mmHg and/or a diastolic blood pressure (DBP) in the range of 80 mmHg to 89 mmHg] are randomized to one of two intervention groups in a 1:1 ratio: Tai Chi or aerobic exercise. BP monitoring methods of office blood pressure, ambulatory blood pressure monitoring (ABPM), and home blood pressure monitoring (HBPM) are used at the same time to detect BP in multiple dimensions. The primary outcome is the comparison of SBP change from baseline to 12 months in Tai Chi group and SBP change from baseline to 12 months in aerobic exercise group. The secondary endpoints are as following: (1) the comparison of DBP of office blood pressure change from baseline to 12 months between Tai Chi group and aerobic exercise group, (2) the comparison of BP and the variability of BP assessed through ABPM change from baseline to 12 months between Tai Chi group and aerobic exercise group, (3) the comparison of BP assessed through HBPM change from baseline to 12 months between Tai Chi group and aerobic exercise group.

**Discussion:**

This will be the first randomized controlled trial to specifically study the benefits of Tai Chi on the blood pressure control in patients with prehypertension. The successful completion of this study will help to provide evidence for whether Tai Chi is more desirable than aerobic exercise.

**Trial registration:**

Trial registration number: Chinese Clinical Trial Registry, ChiCTR1900024368. Registered on 7 July 2019, http://www.chictr.org.cn/edit.aspx?pid=39478&htm=4

**Supplementary Information:**

The online version contains supplementary material available at 10.1186/s13063-022-06840-6.

## Administrative information

SPIRIT 2013 Checklist: Recommended items to address in a clinical trial protocol and related documents*

**Table:** 

Section/item	Item No	Description	Reported on page No
**Administrative information**			
Title	{1}	Descriptive title identifying the study design, population, interventions, and, if applicable, trial acronym	1
Trial registration	{2a}	Trial identifier and registry name. If not yet registered, name of intended registry	3
	{2b}	All items from the World Health Organization Trial Registration Data Set	3
Protocol version	{3}	Date and version identifier	18
Funding	{4}	Sources and types of financial, material, and other support	19
Roles and responsibilities	{5a}	Names, affiliations, and roles of protocol contributors	1, 20
	{5b}	Name and contact information for the trial sponsor	19
	{5c}	Role of study sponsor and funders, if any, in study design; collection, management, analysis, and interpretation of data; writing of the report; and the decision to submit the report for publication, including whether they will have ultimate authority over any of these activities	19
	{5d}	Composition, roles, and responsibilities of the coordinating centre, steering committee, endpoint adjudication committee, data management team, and other individuals or groups overseeing the trial, if applicable (see Item 21a for data monitoring committee)	15, 16
Introduction			
Background and rationale	{6a}	Description of research question and justification for undertaking the trial, including summary of relevant studies (published and unpublished) examining benefits and harms for each intervention	4, 5
	{6}	Explanation for choice of comparators	4, 5
Objectives	{7}	Specific objectives or hypotheses	5
Trial design	{8}	Description of trial design including type of trial (eg, parallel group, crossover, factorial, single group), allocation ratio, and framework (eg, superiority, equivalence, noninferiority, exploratory)	5, 6
Methods: Participants, interventions, and outcomes			
Study setting	{9}	Description of study settings (eg, community clinic, academic hospital) and list of countries where data will be collected. Reference to where list of study sites can be obtained	5
Eligibility criteria	{10}	Inclusion and exclusion criteria for participants. If applicable, eligibility criteria for study centres and individuals who will perform the interventions (eg, surgeons, psychotherapists)	6, 7
Interventions	{11a}	Interventions for each group with sufficient detail to allow replication, including how and when they will be administered	8-10
	{11b}	Criteria for discontinuing or modifying allocated interventions for a given trial participant (eg, drug dose change in response to harms, participant request, or improving/worsening disease)	8-10
	{11c}	Strategies to improve adherence to intervention protocols, and any procedures for monitoring adherence (eg, drug tablet return, laboratory tests)	8-10
	{11d}	Relevant concomitant care and interventions that are permitted or prohibited during the trial	12
Outcomes	{12}	Primary, secondary, and other outcomes, including the specific measurement variable (eg, systolic blood pressure), analysis metric (eg, change from baseline, final value, time to event), method of aggregation (eg, median, proportion), and time point for each outcome. Explanation of the clinical relevance of chosen efficacy and harm outcomes is strongly recommended	13
Participant timeline	{13}	Time schedule of enrolment, interventions (including any run-ins and washouts), assessments, and visits for participants. A schematic diagram is highly recommended (see Figure)	see Figure 2
Sample size	{14}	Estimated number of participants needed to achieve study objectives and how it was determined, including clinical and statistical assumptions supporting any sample size calculations	7, 8
Recruitment	{15}	Strategies for achieving adequate participant enrolment to reach target sample size	6
**Methods: Assignment of interventions (for controlled trials)**			
Allocation:			
Sequence generation	{16a}	Method of generating the allocation sequence (eg, computer-generated random numbers), and list of any factors for stratification. To reduce predictability of a random sequence, details of any planned restriction (eg, blocking) should be provided in a separate document that is unavailable to those who enrol participants or assign interventions	7
Allocation concealment mechanism	{16b}	Mechanism of implementing the allocation sequence (eg, central telephone; sequentially numbered, opaque, sealed envelopes), describing any steps to conceal the sequence until interventions are assigned	7
Implementation	{16c}	Who will generate the allocation sequence, who will enrol participants, and who will assign participants to interventions	7
Blinding (masking)	{17a}	Who will be blinded after assignment to interventions (eg, trial participants, care providers, outcome assessors, data analysts), and how	7
	{17b}	If blinded, circumstances under which unblinding is permissible, and procedure for revealing a participant’s allocated intervention during the trial	7 Only outcome assessors are blinded so unblinding will not occur.
**Methods: Data collection, management, and analysis**			
Data collection methods	{18a}	Plans for assessment and collection of outcome, baseline, and other trial data, including any related processes to promote data quality (eg, duplicate measurements, training of assessors) and a description of study instruments (eg, questionnaires, laboratory tests) along with their reliability and validity, if known. Reference to where data collection forms can be found, if not in the protocol	10, 11 Data collection form is submitted.
	{18b}	Plans to promote participant retention and complete follow-up, including list of any outcome data to be collected for participants who discontinue or deviate from intervention protocols	8-10
Data management	{19}	Plans for data entry, coding, security, and storage, including any related processes to promote data quality (eg, double data entry; range checks for data values). Reference to where details of data management procedures can be found, if not in the protocol	12
Statistical methods	{20a}	Statistical methods for analysing primary and secondary outcomes. Reference to where other details of the statistical analysis plan can be found, if not in the protocol	14, 15
	{20b}	Methods for any additional analyses (eg, subgroup and adjusted analyses)	Not planned
	{20c}	Definition of analysis population relating to protocol non-adherence (eg, as randomised analysis), and any statistical methods to handle missing data (eg, multiple imputation)	15
**Methods: Monitoring**			
Data monitoring	{21a}	Composition of data monitoring committee (DMC); summary of its role and reporting structure; statement of whether it is independent from the sponsor and competing interests; and reference to where further details about its charter can be found, if not in the protocol. Alternatively, an explanation of why a DMC is not needed	16
	{21b}	Description of any interim analyses and stopping guidelines, including who will have access to these interim results and make the final decision to terminate the trial	16
Harms	{22}	Plans for collecting, assessing, reporting, and managing solicited and spontaneously reported adverse events and other unintended effects of trial interventions or trial conduct	13, 14
Auditing	{23}	Frequency and procedures for auditing trial conduct, if any, and whether the process will be independent from investigators and the sponsor	16
Ethics and dissemination			
Research ethics approval	{24}	Plans for seeking research ethics committee/institutional review board (REC/IRB) approval	19
Protocol amendments	{25}	Plans for communicating important protocol modifications (eg, changes to eligibility criteria, outcomes, analyses) to relevant parties (eg, investigators, REC/IRBs, trial participants, trial registries, journals, regulators)	16
Consent or assent	{26a}	Who will obtain informed consent or assent from potential trial participants or authorised surrogates, and how (see Item 32)	6
	{26b}	Additional consent provisions for collection and use of participant data and biological specimens in ancillary studies, if applicable	N/A
Confidentiality	{27}	How personal information about potential and enrolled participants will be collected, shared, and maintained in order to protect confidentiality before, during, and after the trial	14
Declaration of interests	{28}	Financial and other competing interests for principal investigators for the overall trial and each study site	19
Access to data	{29}	Statement of who will have access to the final trial dataset, and disclosure of contractual agreements that limit such access for investigators	20
Ancillary and post-trial care	{30}	Provisions, if any, for ancillary and post-trial care, and for compensation to those who suffer harm from trial participation	12
Dissemination policy	{31a}	Plans for investigators and sponsor to communicate trial results to participants, healthcare professionals, the public, and other relevant groups (eg, via publication, reporting in results databases, or other data sharing arrangements), including any publication restrictions	17
	{31b}	Authorship eligibility guidelines and any intended use of professional writers	20
	{31c}	Plans, if any, for granting public access to the full protocol, participant-level dataset, and statistical code	15
Appendices			
Informed consent materials	{32}	Model consent form and other related documentation given to participants and authorised surrogates	These are available from the corresponding author on request.
Biological specimens	{33}	Plans for collection, laboratory evaluation, and storage of biological specimens for genetic or molecular analysis in the current trial and for future use in ancillary studies, if applicable	N/A

* It is strongly recommended that this checklist be read in conjunction with the SPIRIT 2013 Explanation & Elaboration for important clarification on the items. Amendments to the protocol should be tracked and dated. The SPIRIT checklist is copyrighted by the SPIRIT Group under the Creative Commons “Attribution-NonCommercial-NoDerivs 3.0 Unported” license

## Background

Prehypertension, defined as blood pressure (BP) in the range of 120–139/80–89 mmHg, was introduced by the seventh report of the Joint National Committee on Prevention, Detection, Evaluation, and Treatment of High Blood Pressure in 2003 [1]. Prehypertension is highly prevalent and affects 25–50% of adults worldwide. Compared with optimal BP, the prehypertension increases the risk of incident hypertension, cardiovascular (CV) events, and death [2], as it is related to risk factors such as high body mass index, metabolic syndrome, dyslipidemia, and impaired glucose metabolism [3, 4]. Prehypertension increases the risk of incident hypertension, with annual rates ranging from 8 to 20% in studies lasting 2,4 years and 4 to 9% in longer-term studies [2, 5]. The Guidelines recommend that prehypertension population at low–moderate CV risk should be offered lifestyle advice [6]. Moderate intensity of regular physical activity, as one of the lifestyles, can reduce BP, as well as lower the risk of heart attack and stroke [7].

Aerobic exercise is recommended as one of the lifestyles that can lower BP [8] and suggested in the guidelines to help both the prevention and treatment of hypertension [6]. However, aerobic exercise has some limitations. The exercise adherence is low and pace and venue also restrict effective training of aerobic exercise. Other useful substitute exercise modes need to be proved effective in reducing BP. As a safe, low-impact, enjoyable, and inexpensive form of exercise that requires minimal equipment and space, Tai Chi is beneficial to improve exercise adherence and is expected as a viable alternative to aerobic exercise [9, 10]. Tai Chi guides people to concentrate on slow and fluid movements, covering all-round adjustments such as balance, core strength, and flexibility [11]. Studies have shown that Tai Chi has a positive effect on patients with cardiovascular diseases (CVD) [12], and it can have a beneficial impact on BP when combined with other lifestyle forms changes [13]. Some studies have shown that after 12 weeks, 9 months, or 12 months of intervention, systolic blood pressure (SBP) in the Tai Chi group decreased significantly, suggesting that Tai Chi exercise training can effectively reduce BP in patients with hypertension [14–17].

Therefore, the study aimed to assess the effect of Tai Chi intervention program, compared with aerobic exercise, on the office BP, ambulatory blood pressure monitoring (ABPM) and home blood pressure monitoring (HBPM) in patients with prehypertension. Based on previous studies, Tai Chi was hypothesized to have better effects on lowering BP than aerobic exercise.

## Methods/design

### Study design

**Fig. 1:**
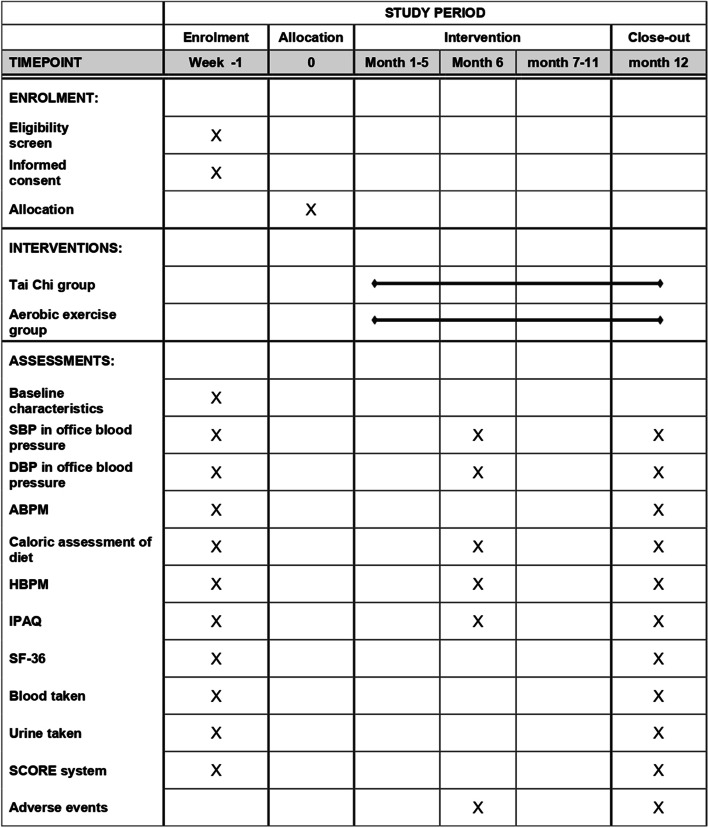
SPIRIT figure. Schedule of enrolment, interventions, and assessments

This protocol followed the Standard Protocol Items: Recommendations for Interventional Trials (SPIRIT) reporting guideline (Fig. 1 and Additional File 1). The study setting is two hospitals in Beijing, China: Guang’anmen Hospital of China Academy of Chinese Medical Sciences and Dongzhimen Hospital of Beijing University of Chinese Medicine. The TCOBPP study has received approval from the Ethics Committee of two hospitals.

This study is a 12-month, parallel, randomized controlled trial, conducted in two centers. Patients with prehypertension are randomized to one of two intervention groups: 12-month supervised Tai Chi held four times a week or 12-month supervised aerobic exercise. All groups will be followed up for 12 months.

**Fig. 2:**
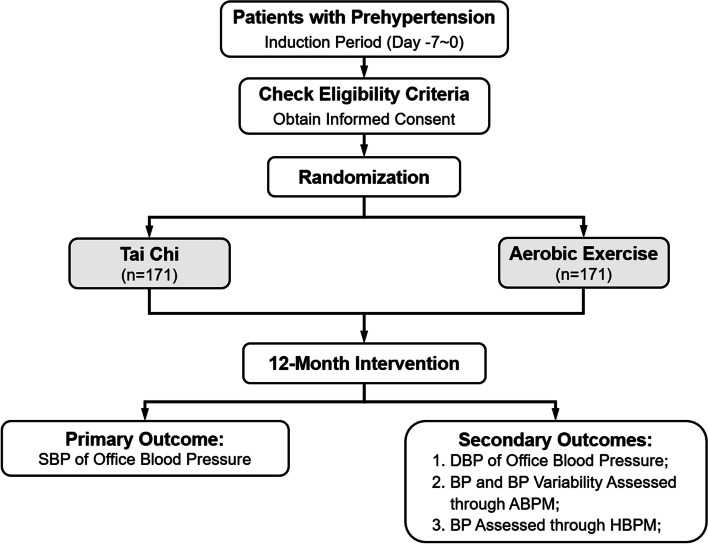
Study flow chart. SBP, systolic blood pressure; DBP, diastolic blood pressure; BP, blood pressure; ABPM, ambulatory blood pressure monitoring; HBPM, home blood pressure monitoring

A 1-week induction period is designed for those who meet the inclusion criteria for prehypertension screening; during this period, participants will receive Tai Chi education and train to exclude individuals who cannot tolerate the exercises and improve study compliance. Outcome measurements are collected at baseline, 6 months, and 12 months (Table 1). The staff conducting the BP assessments and the statistician are blinded to treatment tasks and groupings. The study flow chart shows an overview of the study procedure (Fig. 2).

**Table 1:** Sequence of primary and secondary outcomes measurement during intervention and follow-up

	Baseline	Month 6	Month 12
Time (days)	− 7 ~ 0	180 ± 14	360 ± 14
Primary outcome variable			
SBP in office blood pressure^a^	×	×	×
Secondary outcome variables			
DBP in office blood pressure	×	×	×
ABPM^b^	×		×
Caloric assessment of diet^c^	×	×	×
HBPM	×	×	×
IPAQ^d^	×	×	×
SF-36	×		×
Blood taken	×		×
Urine taken	×		×
SCORE system	×		×

*Abbreviations*: *DBP* Diastolic blood pressure, *HBPM* Home blood pressure monitoring, *IPAQ* International Physical Activity Questionnaire, *SF-36* Medical Outcome Survey Short-Form 36, *SCORE* Systematic COronary Risk Evaluation

^a^ Systolic blood pressure (SBP) in office blood pressure is the primary outcome at 12 months; the other collection times are secondary outcome variables

^b^ Ambulatory blood pressure monitoring (ABPM) can provide the average blood pressure estimates during the whole monitoring period and provide average BP during nighttime and daytime respectively, estimate the variability of BP

^c^ Assess the average daily calorie intake of participant for the nearly week

^d^ Used to assess the one-week total physical activity including physical activity of occupation, transportation, housework, and recreation

### Eligibility criteria

Patients who meet the classification of prehypertension [with a SBP in the range of 120 mmHg to 139 mmHg and/or a diastolic blood pressure (DBP) in the range of 80 mmHg to 89 mmHg] are eligible to participate in this study [1]. Recruitment strategies include the distribution of flyers within the hospital as well as advertisements in print and online media, to ensure adequate enrollment of different study population. Potential participants will be contacted by phone and WeChat to assess whether they meet the basic eligibility criteria for the study. Those who meet the eligibility criteria are invited to come to the hospital for screening, in which case their eligibility criteria are verified. After a complete explanation of the study procedures, each eligible subject who agrees to participate provides informed consent, which is completed by the principal investigator or study coordinator. The study coordinator will inform participants of the schedule of the training sessions, including the date and time.

Participants are considered eligible for this study if they (1) are aged from 18 to 65 years; (2) fulfill the classification of prehypertension: with a SBP in the range of 120 to 139 mmHg and/or a DBP in the range of 80 mmHg to 89 mmHg [1]; (3) have no western medicine or traditional Chinese medicine, acupuncture and moxibustion were used to control blood pressure (or the treatment was discontinued for 2 weeks); (4) are willing to be randomized to Tai Chi group or aerobic exercise group; (5) have ability to complete written questionnaires and operate electronic equipment independently; and (6) are able to give informed consent. Exclusion criteria are (1) diagnosed with diabetes mellitus and coronary heart disease; (2) pregnant and lactating women; (3) non-dominant arm circumference > 50 cm; (4) body mass index (BMI) larger than 40.0 kg/m^2^; (5) take benzodiazepines, antipsychotics, or oral glucocorticoids (allowed to taken fluoxetine, paroxetine, sertraline, fluvoxamine, citalopram and escitalopram stably within 3 months); (6) with chronic kidney disease, with eGFR < 60 mL/min; (7) diagnosed with Shy-Drager syndrome; (8) alcoholism (male’s alcohol intake is more than 25 g/day or 140 g/week or female’s alcohol intake is more than 15 g/day or 80 g/week); (9) has played Tai Chi more than once a month in the past 6 months; (10) plays vigorous sports activities more than three times a week; (11) with musculoskeletal disorder or other disabling diseases lead to the inability to practice Tai Chi or do aerobic exercise; and (12) current in clinical trials of other drugs or external therapies.

### Randomization and blinding

Participants, after the induction period, who met all the eligibility criteria and provided written informed consent, will be randomly assigned to either Tai Chi or aerobic exercise (control) group, in a 1:1 ratio, with a predicted sample size of 342 patients, 171 in each group. At the same time, the participants are stratified according to SBP of 120–129 mmHg and DBP < 80 mmHg and SBP of 130–139 mmHg or DBP of 80–89 mmHg. At each stratum, the proportion of participants in the Tai Chi and aerobic exercise groups is also randomly assigned in a 1:1 ratio. In order to ensure the concealment of allocation, a 24-h central web-based automated randomization system is adopted for all randomization processes, using the static random method and the SAS9.4 software PROC PLAN process programming.

When allocation is complete, the outcome assessors who evaluate the effects of the treatments will receive only the participant number, and then interpretation the data under blinded to group allocation. Only outcome assessors are blinded so unblinding will not occur.

### Sample size

The sample size calculation was based on the comparison of the office SBP drop of individuals in the Tai Chi group and the aerobic exercise group. According to the average reduction of SBP in the studies conducted before the start of the trial [14, 18], using a conservative estimate, we hypothesized that the SBP in the Tai Chi group would be reduced by 4.6 mmHg more than the aerobic exercise group. Further conservatively assuming an SD of 13.4 of both groups. We estimate a loss of follow-up of 20% and 80% power at a two-sided α level of 0.05. Therefore, 171 participants per group and 342 participants in total is the reasonable sample size of this study. The analyses were performed using PASS version 15.0.

### Study intervention

The maximum waiting time between baseline assessment and interventional therapy is 3 weeks. In order to avoid the influence of seasonal factors on the disease, both Tai Chi and aerobic exercise groups are simultaneously performed. Participants randomly assigned to Tai Chi or aerobic exercise will practice at indoor activity room in or near Guang’anmen Hospital or Dongzhimen Hospital. The number of participants in each class is limited to 20 to ensure the quality of teaching and learning.

#### Lifestyle intervention

Common interventions are health education and lifestyle guidance. Participants in both groups receive dietary recommendations for weight control and salt intake. DASH eating plan is the best diet that can effectively reduce BP [19]. During the study intervention, participants are advised to follow the DASH diet, which is rich in fruits, vegetables, and low-fat dairy foods and with reduced saturated and total fat.

#### Tai Chi intervention

The 24-form Yang-style Tai Chi consists of 24 standard movements. The four Tai Chi instructors each have extensive experience and will explain and demonstrate Tai Chi principles, practice techniques, and safety precautions for each movement at the beginning of the study. The instructor will review these principles and techniques, as throughout the study process and always practice with the participants, to timely and effectively identify and correct the incorrect posture or movement. Participants will also be instructed to concentrate and perform traditional Tai Chi breathing, while performing body movements. Moreover, all four instructors completed the required human subject protection training before the beginning of intervention courses.

Each Tai Chi session will last for 60 min, including 10 min of warm-up exercise, 40 min of Tai Chi teaching and/or practice, and 10 min of relaxation and occur 4 times a week. Among them, there are no less than twice centralized sessions per week, and for the rest practice, participants can practice at home and upload videos. The instructor could modify and tailor-make for their Tai Chi exercises according to participants' learning and athletic ability. In the initial eighth week, the participants learn and practice step by step. In each session, participants practice and learn 3 to 4 movements of Tai Chi. After all the 24 Tai Chi forms had been learned (weeks 10 and 11), the Tai Chi instructor (experience > 10 years) will assess the participants. After passing the assessment, the participants will participate in centralized sessions at least once a week and practice at home and upload videos for the other three times. Participants are required to sign in to confirm the accurate attendance records, when they attend the Tai Chi session or practice at home. The standard case report forms are used to record and verify the data collected for class attendance, to confirm accurate attendance recordings. The study staff will monitor the participants by monthly home calls throughout the 12-month intervention. Throughout the study, all sessions are regularly monitored and fed back to ensure proper instruction.

#### Aerobic exercise intervention

Participants randomized to aerobic exercise will receive a supervised, group-format aerobic exercise program. The aerobic exercises training protocol for prehypertension treatment consists of four 60-min sessions of moderate intensity exercises per week. The aerobic exercises include climbing stairs, jogging, brisk walking, and cycling. Each session includes several parts: 10 min of warm-up including low-intensity exercise and dynamic stretching; 40 min of organized aerobic training, gradually developing from low intensity to medium intensity; and 10 min cooldown. The training in the sessions is progressive, and all participants gradually increase the duration and intensity of the exercise. In all sessions, instructors will closely monitor to ensure the comfort and safety of participants and to minimize adverse events. Heart rate will be recorded during each session to monitor the intensity of exercise. During the 1–4 weeks, participants are advised to achieve an individualized heart rate of 55%—–65% of estimated maximum heart rate according to their age and should reach 60–70% after 4 weeks. The maximum heart rate is estimated as “208 − 0.7 × age” [20]. Participants in aerobic exercise group will perform the above exercises 4 times a week, including collective exercises no less than 1 time a week, and the rest 3 times of uploaded videos. A wrist wearable device (HUAWEI band) is used to monitor heart rate. It uses an optical sensor to accurately calculate heart rate through fluctuations in blood flow in the wrist. The data are uploaded to an app, where information is stored for long time. The data collected for session attendance are recorded using standard case report forms. The study team will contact the participants by monthly phone to monitor their adherence until the 12-month follow-up evaluation. During the study process, all sessions are regularly monitored by the instructors and fed back to ensure correct instruction for the group.

### Measurements

Referring to the BP measurement method in the guideline [21], the specific measurement method of the study is as follows. A quiet room is set up for measurement of BP. The upper arm medical electronic sphygmomanometer certified by the internationally accepted protocol is used (Omron HBP-1300). The patients are asked to rest, sitting in a chair, for > 10 min, and the first BP measurement is conducted following the rest period. The participant and the researcher should not talk during the rest period or the measurement. The interval of repeated measurements is 1–2 min. When the difference between the first two measurements is greater than 10 mmHg, additional measurements are taken. The average of the last two readings is recorded to estimate the individual’s level of BP.

Participants receive 24 h-ambulatory blood pressure monitoring (Welch Allyn ABPM 6100). The 24-h ABPM is programmed to automatically obtain BP records, with the instrument set to obtain readings every 30 min throughout the day and every 1 h at night. ABPM can provide the average BP estimates during the whole monitoring period and provide average BP during nighttime and daytime, respectively, and estimate the variability of BP.

Participants are given a free upper-arm cuff device (Lifesense i5S), which enables automatically stores multiple readings, and are educated on its use. Referring to the home BP monitoring measurement in the guideline [6], measurements are taken in a quiet room after 5 min of rest, with the participant seated. Participants are instructed to obtain home BP measurements two times in the morning after voiding and before eating or vigorous exercise and two times in the evening, with 1 min apart between readings. Mean home BP is reliable; using the average of two morning and two evening BP readings for at least three consecutive days in 1 week minimum in each month [22], at the same time, can be used to estimate the variability of BP.

### Data collection and management

#### Plans for assessment and collection of outcomes

The researchers shall fill in data to case report forms (CRF) accurately, completely, and timely based on original observations of the subjects. The auditor should monitor whether all CRFs are consistent with the source data and raise questions at any time when any problem occurs. If there are errors and omissions, the researchers should correct them in time.

#### Data management

All data will initially be entered legibly in the paper CRF. If an error is made, it will be crossed through with a single line to ensure that the original entry can still be read. The correct entry will then be inserted clearly. The amendment will be initialed and dated by the person making the correction immediately. It is not permitted to overwrite or use correction fluid. Ensure that the paper-based CRF data are securely input into Electronic Data Capture (EDC) system. Access will be restricted to site personnel, trial monitors, and data management team. The EDC system provides range checks for data values to ensure and improve data quality. Researchers are responsible for ensuring the accuracy of all data entered and recorded in the paper CRF and EDC system.

#### Relevant concomitant care permitted or prohibited during the trial

During the run-in period and the intervention period, antihypertensive drugs or antihypertensive treatment will not be allowed to be used. If the participant has increased BP and cannot tolerate the increased BP, which should be treated immediately, it is not advisable to continue the trial, and the researcher should consider terminating the intervention and switching to another type of clinical treatment.

#### Provisions for post-trial care

Participants will be assessed on a case-by-case basis across all participating sites and will be given the option to remain on the centralized exercise training after completion of the trial if there appears to be a benefit to them. If serious adverse events occur during the study period, the participants are also required to be followed up after the study period. Appropriate measures will be taken to fully protect the interests of participants, such as outpatient or inpatient care or referrals to other specialists.

### Outcomes and follow-up

At enrollment, the essential information of participants’ sex, age, nation, income, education level, professional characteristics, personal history, family history, height, weight, waist circumference, hip circumference, eating habits, and caloric intake are collected. Overall assessment of BP is focused on. Outcomes are assessed at baseline, 6 months, and 12 months (at the end of the intervention).

#### Primary outcome

The primary outcome is the comparison of SBP of office blood pressure change from baseline to 12 months between Tai Chi group and aerobic exercise group.

#### Secondary outcomes

BP monitoring methods of office blood pressure, ABPM, and HBPM are used at the same time to detect BP in multiple dimensions. The secondary endpoints are as following:

1. The comparison of DBP of office blood pressure change from baseline to 12 months between Tai Chi group and aerobic exercise group
2. The comparison of BP and the variability of BP assessed through ABPM change from baseline to 12 months between Tai Chi group and aerobic exercise group
3. The comparison of BP assessed through HBPM change from baseline to 12 months between Tai Chi group and aerobic exercise group

### Monitoring of adverse events

Adverse events must be registered during the study period, which refers to the medical conditions not considered as end points of study. Study participants are monitored for the occurrence of adverse events, during each encounter during the study intervention. A study telephone number is provided to the participants to report adverse events throughout the study. All adverse events are recorded in the case report form during the study intervention.

### Criteria for study withdrawal

Individuals who decide to no longer participate in the study or who are lost to follow-up (failure to attend the prescheduled visits or cannot be reached by telephone call) can be withdrawn from the study.

### Confidentiality

Participant individual identification numbers are used to track data collection documents. All data will be kept strictly confidential and only accessed by members of the trial team. Paper CRFs are stored in a locked file cabinet. Access to EDC system is password protected and restricted to the trial team. This protocol, CRFs, and other documents and materials related to the trial will be kept strictly confidential and will not be disclosed to third parties unless expressly agreed upon by the principal investigator in advance. Staff of the investigators involved in this trial are also bound by the agreement.

### Statistical analysis

The data of all participants who completed the follow-up will be analyzed according to the group to which they were originally assigned, regardless of whether having adhered to the treatment and study procedures. Descriptive statistics, such as the mean (SD) or percentage, are used to summarize baseline characteristics and unadjusted study outcome measures, while assessing intergroup equivalence at baseline. At the end of the study, treatment analysis will be performed according to the protocol. Analysis of variance of continuous variables and *χ*^2^ (or Fisher exact) test for categorical variables are used to compare baseline demographic descriptions and primary and secondary outcome measures of each group. For continuous variables, the differences between the average variations from baseline values and their respective 95% CIs will be calculated. If any characteristic is substantially different at baseline, it will be adjusted in a mixed-model regression analysis. To avoid multiple comparisons, differences between groups will be assessed only if the overall effect of treatment is significant. The source data will be registered in the paper CRF and EDC system, and the pattern of missing data will be evaluated before data analyzing. Missing data will be processed through multiple imputation using a set of baseline characteristics and 6-month and 12-month results. All analyses will be conducted using IBM SPSS (IBM Corp) or Stata (release 13; StataCorp LP). Level of statistical significance will be set at 0.05.

### Plans to give access to the full protocol, participant-level data

Detailed information of the trial including study design, eligibility criteria, and outcome measures are available to the public on Chinese Clinical Trial Registry, ChiCTR1900024368. The data analyzed during the current study are available from the corresponding author on reasonable request.

### Oversight and monitoring

The study group provided coordination and day to day support for the trial. The study leader, Yanwei Xing, supervised the design of the study and will supervise and guide the implementation of the trial. Study leader was responsible for all aspects of local organization, including identifying potential recruits and taking consensus. The trial steering committee is composed of the study leader (Xing), on-site principal investigators of each hospital, and coinvestigators. Questions that arise during the research process will be submitted to the committee for decision-making. Finally, clinical research associates (CRAs) will supervise the study progress at any time and hold a meeting every three months.

The Data Monitoring Committee (DMC) consists of a doctor whose major is clinical cardiovascular disease, a scientific researcher whose major is clinical trial methodology, and a statistician. DMC is responsible for safety monitoring, reviewing, and evaluating the detailed information of adverse events between groups. According to the evaluation results, DMC could give suggestions to terminate the trial in advance or take measures to reduce the risk of adverse events and adjust the study protocol. In the middle of the trial, the research team will conduct interim analysis. If the analysis result is consistent with the hypothesis, the test will be continued. If it is inconsistent or even contrary, the expert committee will be consulted to decide whether to continue the test, expand the sample size, or terminate the test.

### Plans for auditing trial conduct

The monitoring will be conducted by the principal investigator, on-site principal investigators, and DMC every 3 months, as an audit of trial conduct. Annual progress reports and interim report are provided to the funding agent.

### Plans for communicating important protocol amendments

Any changes to the protocol will notify the funding agent first then will notify the centers and that a copy of the revised protocol will be added to the Investigator Site File. All subsequent substantial protocol amendments will be documented and submitted to the Ethics Committee for approval before implementation. The principal investigator at each site is responsible for ensuring that all subsequent amendments gain the necessary approval.

### Dissemination plans

The results of this study will be widely disseminated through a series of peer-reviewed publications; presentations at local, national, and international academic conferences and reports to funding agent. In addition, a summary of the primary outcome findings will be created in English and Chinese and shared with the study participants.

## Discussion

As far as we know, this is the first randomized controlled trial to specifically study the benefits of Tai Chi on the blood pressure control in patients with prehypertension. Previous study has suggested that prehypertension patients have a twofold to threefold higher risk to develop hypertension than those with normal BP [2]. In a 2017 study, Kanegae et al. summarized that the 5-year rates of hypertension in the population with high-normal blood pressure can reach 50% [23]. Being in the prehypertension period for a long time not only has a significant influence on the cardiovascular system but also easily develops clinical hypertension.

The guidelines showed that effective and healthy lifestyle choices can prevent or delay the occurrence of hypertension and reduce CV risk [6]. Physical activity and exercise are the cornerstone of blood pressure control and reducing in some case CV risk, especially before entering the stage of hypertension. Tai Chi, as a meditative exercise, can cause internal functional balance and enhance physiological and psychological functions, so as to promote healing, stress neutralization, and personal harmony [24]. Previous studies have shown that Tai Chi led to effective blood pressure reduction in the short or long term [14–17]. Tai Chi exercise may be an effective method of hypertension, especially for patients who are in the stage of prehypertension. It has previously been demonstrated to have a positive effect on blood pressure control. A previous meta-analysis of the effects of Tai Chi on essential hypertension in Chinese adults showed that Tai Chi can reduce the SBP and DBP of hypertensive patients [25]. However, further studies are needed to evaluate the effectiveness of Tai Chi in the treatment of prehypertension. It is worth noting that, in our study, blood pressure monitoring methods of office BP, ABPM, and HBPM are used at the same time to detect BP in multiple dimensions and get more comprehensive and multi angle results.

In order to further explore the effect of Tai Chi exercise on prehypertension, this study aims to provide more convincing and detailed information on Tai Chi for better blood pressure control. The successful completion of this study will help to provide evidence for whether Tai Chi is more desirable than aerobic exercise. Through this study, with a large sample and long term of intervention and follow-up (12 months), conducted in two centers, to understand the benefits of Tai Chi in reducing CVD risk factors is of great value to guide the formulation of effective intervention to have an important impact on the public health of prehypertension patients.

### Trial status

The current protocol is version 5.1, dated 10 September 2020. Participant recruitment and randomization began in September 2019. Recruitment has been completed in January 2021.

## Supplementary Information

**Additional file 1.** SPIRIT 2013 Checklist: Recommended items to address in a clinical trial protocol and related documents.

## Data Availability

Not applicable as data is not yet available.

## Abbreviations

ABPM: Ambulatory blood pressure monitoring
BMI: Body mass index
BP: Blood pressure
CRA: Clinical research associates
CRF: Case report forms
CV: Cardiovascular
CVD: Cardiovascular diseases
DBP: Diastolic blood pressure
DMC: Data Monitoring Committee
EDC: Electronic Data Capture
HBPM: Home blood pressure monitoring
SBP: Systolic blood pressure
